# Association between neutrophil-lymphocyte ratio and female breast cancer: an observational study from NHANES 2001-2018 with external validation

**DOI:** 10.3389/fonc.2025.1564238

**Published:** 2025-07-08

**Authors:** Juan Xiong, Deju Zhang, Jing Wu, Peili Yang, Donghai Shi, Xiaolan Zhou, Ying Yuan, Chuntao Quan, Ni Xie

**Affiliations:** ^1^ BioBank, Shenzhen Second People’s Hospital, Heng Yang Medical School, University of South China, Hengyang, Hunan, China; ^2^ BioBank, Shenzhen Second People’s Hospital, the First Affiliated Hospital of Shenzhen University, Shenzhen, China; ^3^ Department of Family Medicine, The Second People's Hospital of Deyang, Deyang, Sichuan, China; ^4^ Department of Infectious Diseases, Peking University Shenzhen Hospital, Shenzhen, China; ^5^ The Fourth Clinical Medical College of Guangzhou University of Traditional Chinese Medicine, Shenzhen, China

**Keywords:** neutrophil-lymphocyte ratio, NHANES, female breast cancer, AIC/BIC, cross-sectional study

## Abstract

**Background:**

In the 21st century, breast cancer is the most frequent malignant tumor threatening women’s health. Previous research has confirmed that inflammatory response processes play key roles in tumor occurrence, development, and metastasis. The neutrophil-to-lymphocyte ratio (NLR), an emerging disease biomarker, has become a focus of cancer research. However, analysis of the relationship between NLR and breast cancer remains scarce. Therefore, our study explored NLR levels in relation to female breast cancer (FBC) prevalence.

**Methods:**

We analyzed data from 15,313 adult females aged 20 and above, using the 2001 to 2018 National Health and Nutrition Examination Survey (NHANES). We explored the association between NLR and FBC prevalence using multiple statistical approaches, including descriptive analysis, multivariate logistic regression, and subgroup analyses. We applied Akaike Information Criterion (AIC) and Bayesian Information Criterion (BIC) to measure model performance. Additionally, smooth curve fitting examined the potential non-linear relationship. To validate our findings, an independent external validation dataset comprising 250 participants (50 breast cancer cases and 200 controls) from Shenzhen Second People’s Hospital was utilized, and correlation between NLR values and breast cancer prevalence was calculated.

**Results:**

NLR was positively associated with FBC prevalence among US women. In the fully adjusted model, each unit NLR elevation increased FBC prevalence odds by 14% [OR = 1.14 (95% CI: 1.08, 1.22)]. Participants in the highest quartile of NLR had 67% higher FBC prevalence compared to those in the lowest quartile [OR = 1.67 (95% CI: 1.24, 2.24)], with statistical significance across three models at *P* for trend values <0.001. Based on AIC and BIC criteria, multivariable-adjusted models showed superior fit over unadjusted ones for both continuous and categorical NLR specifications. Subgroup analysis showed the positive association between NLR and breast cancer prevalence was consistent across the general population. External validation confirmed robustness, demonstrating positive associations between elevated NLR and breast cancer prevalence.

**Conclusions:**

In the U.S. adult female population, NLR levels were positively correlated with breast cancer prevalence. External validation in Chinese clinical participants supported the generalizability of these findings across different populations.

## 1 Introduction

Globally, approximately 2.3 million new breast cancer cases were reported in 2020, the incidence of breast cancer had exceeded other major malignancies like lung and stomach cancer, and had emerged as the leading cause of cancer mortality in women (1). Integration of molecular-targeted therapies and standardized treatment protocols has markedly improved survival rates in breast cancer patients (2). However, the significant intratumoral heterogeneity and therapeutic resistance remain substantial obstacles for complete tumor eradication, particularly in advanced-stage disease and metastatic settings, leading to considerable mortality rates despite standard-of-care treatment modalities (3, 4). The limitations of existing breast cancer management protocols emphasize the pressing need to develop novel and early detection techniques that not only can diagnose breast cancer at an early stage to prevent disease progression and adverse prognosis, but also address the financial burdens of patients, ultimately aiming to optimize both clinical outcomes and quality of life.

Inflammation, especially chronic inflammation caused by persistent stimulation, involves complex interactions between various immune cells and inflammatory mediators (5). This process is characterized by the persistent activation of inflammatory cells, namely neutrophils, macrophages, and lymphocytes, along with the sustained production of pro-inflammatory cytokines, specifically factor-α (TNF-α), tumor necrosis interleukin-1β (IL-1β), and interleukin-6 (IL-6) (6, 7). These pro-inflammatory cytokines enhance cancer cell survival and proliferation through activation of nuclear factor-κB (NF-κB) and signal transducer and activator of transcription 3 (STAT3) signaling pathways, while neutrophil-derived factors promote angiogenesis and epithelial-mesenchymal transition (EMT) via phosphatidylinositol 3-kinase/protein kinase B (PI3K/AKT) pathway activation (8, 9). Notably, these inflammatory mediators contribute to chemoresistance through multiple mechanisms: primarily, PI3K/AKT pathway activation upregulates the expression of multidrug resistance protein 1 (MDR1), breast cancer resistance protein (BCRP), and multidrug resistance-associated proteins (MRPs), enhancing drug efflux capacity in tumor cells (10, 11). Secondly, persistent inflammatory stimulation induces the expression of anti-apoptotic proteins (such as Bcl-2, Bcl-xL, and Survivin), reducing tumor cell sensitivity to chemotherapy-induced apoptosis (12). Furthermore, elevated reactive oxygen species (ROS) levels in the inflammatory microenvironment activate DNA repair mechanisms, augmenting tumor cell tolerance to DNA-damaging chemotherapeutic agents (13). Prolonged inflammatory stimulation can lead to dysregulation of immune checkpoint molecules, particularly programmed cytotoxic T lymphocyte-associated protein 4 (CTLA-4) and cell death protein-1/programmed death-ligand 1 (PD-1/PD-L1), accompanied by the accumulation of immunosuppressive regulatory T cells (Tregs) in injured areas, resulting in local immune tolerance (14). This immunosuppressive microenvironment compromises immune surveillance, allowing mutated cells to evade recognition and elimination by natural killer (NK) cells and cytotoxic T lymphocytes (15, 16). Subsequently, the proliferation of the mutated cells, combined with chronic inflammation-induced DNA damage and epigenetic alterations, creates a permissive microenvironment for malignant transformation and cancer progression (17). Given the close association between inflammation and cancer, utilizing inflammation-related markers for cancer prediction appears to be a viable strategy. The neutrophil-to-lymphocyte Ratio (NLR) is a calculated value based on the counts of neutrophils and lymphocytes obtained from a routine complete blood count (CBC) test and it has been applied to evaluate the prior-to-treatment equilibrium between inflammatory factors and immune status in people with cancer (18, 19).

The landmark study by Walsh et al. established the NLR applications in colorectal cancer prognosis through a comprehensive analysis of 230 surgical patients. Results revealed that preoperative NLR demonstrated strong predictive capability for survival outcomes, with patients having NLR ≥5 exhibiting significantly reduced 5-year overall survival, thus validating its effectiveness as a prognostic biomarker in colorectal cancer management (20). A previous study suggested that assessing NLR before surgical or medical treatment could serve as an independent prognostic marker of overall survival (OS) and disease-free survival (DFS) in patients with breast cancer (21). However, despite these established associations with cancer prognosis, its potential role as an indicator of breast cancer prevalence in the general population remains unclear. Therefore, we explored the association between NLR and breast cancer utilizing the National Health and Nutrition Examination Survey (NHANES) data between 2001 and 2018, with model fitness evaluated by both Akaike Information Criterion (AIC) and Bayesian Information Criterion (BIC) criteria. Additionally, recognizing the importance of external validation in establishing the robustness of epidemiological findings, we conducted a validation study using an independent clinical dataset to confirm the reproducibility of our results across different populations and healthcare settings.

## 2 Materials and methods

### 2.1 Population and study design

#### 2.1.1 NHANES primary dataset

**Figure 1:**
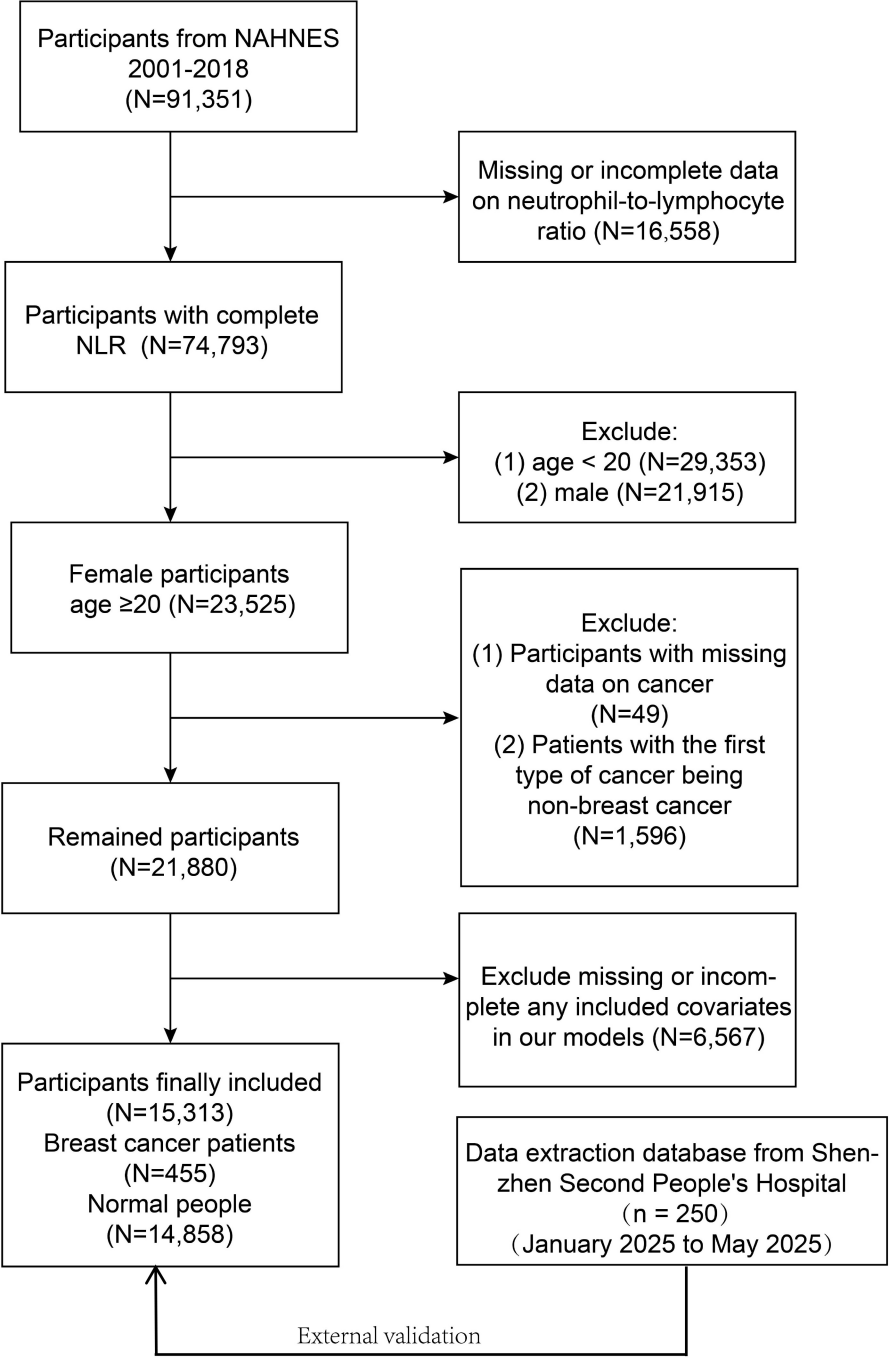
Flow chart of participants selection.

NHANES is a survey of the health and nutrition conditions of the US civilian non- institutionalized population. This database ensures data quality through its comprehensive validation system including cross-validation of self-reported diagnoses with medical records, embedded verification questions, and regular quality control checks. Our study involved data from 91,351 participants for the 9 cycles (2001-2018), of which 76,038 participants were excluded due to incomplete data, and 15,313 participants (455 breast cancer participants and 14,858 normal females) were finally recruited. **Figure 1** presents the full sample exclusion process. All participants informed written consent was given at recruitment, the NCHS Research Ethics Review Board agreed to the study methodology, and no external ethics approval was required to perform the study. The detailed experimental design and data can be accessed at NHANES (https://www.cdc.gov/nchs/nhanes).

#### 2.1.2 External validation dataset

To validate the robustness and generalizability of our findings, the correlation between NLR and breast cancer prevalence was calculated using the data from an independent external validation dataset. This validation samples included patients who underwent routine blood tests and breast cancer screening between January 2025 and May 2025. The similar inclusion and exclusion criteria were applied to ensure comparability with the NHANES participants, specifically including female participants aged 20 years and older with complete blood count data and documented breast cancer status. The validation dataset comprised 250 participants, including 50 breast cancer cases and 200 age-matched controls. Ethical approval for the validation study was obtained from the Institutional Review Board of Shenzhen Second People’s Hospital, and all participants waived written informed consent.

### 2.2 Calculation and assessment of NLR

The NLR is calculated as the ratio of neutrophil count (N) to lymphocyte count (L), expressed as NLR = N/L.

### 2.3 Diagnosis and assessment of breast cancer

This study utilized self-reported cancer diagnoses obtained through medical history questionnaires. Participants were asked to indicate whether they had been diagnosed with cancer by a healthcare professional. Those who responded “No cancer” were classified as controls, while those answering “YES” were prompted to specify their cancer type. Individuals reporting a breast cancer diagnosis were assigned to the breast cancer group. In the validation study, breast cancer cases were identified through routine screening programs at Shenzhen Second People’s Hospital. All suspected cases underwent mammography or ultrasound examination, followed by tissue biopsy when indicated. Breast cancer diagnosis was confirmed by histopathological examination. Only participants with pathologically confirmed breast cancer were included in the case group, while those with normal screening results or benign findings were classified as controls.

### 2.4 Covariates

#### 2.4.1 NHANES dataset

As reported in recent literature (22, 23), Age, race/ethnicity, level of education, marital status, body mass index (BMI), hypertension status, diabetes status, reproductive health factors including age at menarche, pregnancy and oral contraceptive use, as well as smoking history were considered to be important potential covariates. Race/ethnicity was comprised of Non-Hispanic White, Non-Hispanic Black, Mexican American, and Other Race. Education attainment was divided into less than high school, high school graduate, and college or higher. Marital status was grouped into having both a sexual partner and an asexual partner. Hypertension status was categorized as having or not having hypertension. Diabetes status was separated into two categories: no diabetes and diabetes. Smoking history was considered as never, former and current smoker. Finally, the model also contained reproductive health status, which included age at menarche (<12 years or ≥12 years), ever in a pregnancy (no or yes), and oral contraceptive use (no or yes).

#### 2.4.2 External validation dataset

In the validation study, covariates were collected where available and included age, BMI, hypertension status, diabetes status, marital status, smoking history, and pregnancy history. Some covariates from the NHANES analysis (race/ethnicity, education, age at menarche, and oral contraceptive use) were not available in the clinical dataset due to routine data collection practices.

### 2.5 Statistical analyses

All statistical analyses were performed using NHANES sampling and following the Centers for Disease Control and Prevention (CDC) guidelines to account for the complex multistage survey design, data quality assessment was performed with third-party evaluation, which confirmed the appropriateness and robustness of the statistical methodology in addressing the research objectives. Continuous variables were expressed as mean ± standard deviation (SD), whereas categorical variables were expressed as percentages (95% confidence interval, 95% CI). The chi-squared test was used to compare categorical variables between female breast cancer (FBC) patients and normal women, while Student’s t-tests were applied for continuous variables. Logistic regression was used to calculate the covariate-adjusted odds ratio (OR) for the association between NLR and FBC. After the classification of the NLR quartiles, a trend test was performed to assess the trend of linear association between NLR and breast cancer. Model I did not include any covariate adjustments. Model II adjusted for demographic factors, including age, race/enthnity. Model III built upon Model II by additionally adjusting for clinical and reproductive factors, including education level, marital status, BMI, smoking history, hypertension status, diabetes status, pregnancy history, oral contraceptive use, and age at menarche. The goodness of fit was evaluated using AIC/BIC criterion, with lower values suggesting better model fit. Subgroup analyses were performed to investigate the correlations between NLR and breast cancer in women of different ages, education levels, BMI, marital status, diabetes status, and hypertension status, and interaction tests were used to examine the stability of the association between subgroups. The relationship between NLR and breast cancer was clearly presented by a smoothing curve fitting. For the external validation study, statistical analyses were performed using standard methods without survey weights, as the data were collected from a single clinical center. Descriptive statistics and logistic regression analyses followed the same analytical framework as the NHANES study. Given the limited availability of covariates, the validation analysis primarily focused on models adjusted for available clinical variables (age, BMI, hypertension status, diabetes status, smoking history, marital status, and pregnancy history). The goodness of fit was evaluated using AIC/BIC criterion, with lower values indicating better model fit. All analyses were conducted using R software (version 4.3.1), DecisionLinnc (version 1.0), and Empowerstats (version 2.0). *P <*0.05 was considered statistically significant.

## 3 Results

### 3.1 Baseline characteristics of the population

Our study population consisted of 15,313 participants, with a mean ± SD age of 48.59 ± 17.90 years, with FBC patients accounting for 2.97%. Participant baseline characteristics were summarized in **Table 1**. The participants were categorized by ethnicity, Non-Hispanic White participants made up the largest group at 42.67%, followed by Non-Hispanic Black (19.70%), Mexican American (17.35%), and Other Ethnicities (20.28%). The FBC patients were significantly older compared to normal individuals (*P <*0.001) and FBC patients were more prone to be of Non-Hispanic White ethnicity (*P <*0.001). Additionally, compared to the non-breast-cancer group, participants in the breast cancer group were more likely to be without a sexual partner (48.79% vs. 43.30%, *P* = 0.020), ever been pregnant (91.43% vs. 84.83%, *P <*0.001), without oral contraceptive use (42.86% vs. 33.41%, *P <*0.001), former smoker (29.01% vs. 17.78%, *P <*0.001), with a history of diabetes (20.66% vs. 10.53%, *P <*0.001), with a history of hypertension (55.60% vs. 31.82%, *P <*0.001), a lower lymphocyte counts [1.89 ± 0.69 vs. 2.19 ± 0.88, *P <*0.001], and a higher NLR value [2.53 ± 1.58 vs. 2.14 ± 1.12, *P <*0.001]. However, no significant differences were observed between the breast cancer and the non-breast cancer group in education level, age at menarche, BMI, and neutrophil counts.

**Table 1:** Characteristics among female population ≥20 years of age from NHANES 2001–2018 (n =15313).

Characteristics	All participants (n =15313)	Without breast cancer (n = 14858)	With breast cancer (n = 455)	*P*-value
Age at interview, years	48.59 ± 17.90	48.02 ± 17.75	67.34 ± 11.50	<0.001
Race/Ethnicity, %				<0.001
Non-Hispanic White	42.67	42.01	64.18	
Non-Hispanic Black	19.70	19.87	13.85	
Mexican American	17.35	17.61	9.00	
Other Race	20.28	20.51	12.97	
Education level, %				0.115
Below high school	24.03	24.15	20.22	
High school	22.06	22.07	21.76	
College or above	53.91	53.78	58.02	
Marital status, %				0.020
Sexual partner	56.54	56.70	51.21	
Asexual	43.46	43.30	48.79	
Ever been pregnant, %				<0.001
No	14.97	15.17	8.57	
Yes	85.03	84.83	91.43	
Oral contraceptive use, %				<0.001
No	33.69	33.41	42.86	
Yes	66.31	66.59	57.14	
Age at menarche, years				0.841
<12	20.15	20.16	19.78	
≥12	79.85	79.84	80.22	
BMI, kg/m^2^	28.03 ± 5.32	28.03 ± 5.32	28.09 ± 5.26	0.806
Smoking history, %				<0.001
Never	65.11	65.21	61.76	
Former	18.12	17.78	29.01	
Current	16.78	17.01	9.23	
Hypertension, %				<0.001
No	67.47	68.18	44.40	
Yes	32.53	31.82	55.60	
Diabetes, %				<0.001
No	89.17	89.47	79.34	
Yes	10.83	10.53	20.66	
Neutrophil counts,10^3^/µL	4.31 ± 1.75	4.31 ± 1.75	4.20 ± 1.67	0.180
Lymphocyte counts, 10^3^/µL	2.18 ± 0.87	2.19 ± 0.88	1.89 ± 0.69	<0.001
NLR	2.15 ± 1.14	2.14 ± 1.12	2.53 ± 1.58	<0.001

Mean ± SD for continuous variables: the *P*-value was calculated by the Student’s t-test; (%) for categorical variables: the *P*-value was calculated by the chi-square test. NLR, neutrophil-to-lymphocyte ratio; BMI, body mass index.

For external validation, we enrolled 250 female participants aged ≥20 years from Shenzhen Second People’s Hospital, with a mean ± SD age of 52.14 ± 14.45 years (**Table 2**). The validation population included 50 breast cancer patients (20%) and 200 controls (80%). Similar to the NHANES population, breast cancer patients in the validation sample were significantly older than controls (57.54 ± 11.73 vs. 50.80 ± 14.77 years, *P* = 0.003). Consistent with findings from the NHANES population, the validation sample showed that breast cancer patients had significantly lower lymphocyte counts (1.20 ± 0.50 vs. 2.00 ± 0.57, *P <*0.001) and higher NLR values (3.86 ± 2.10 vs. 2.01 ± 0.59, *P <*0.001) compared to controls. Notably, the validation population demonstrated distinct patterns compared to NHANES findings. Breast cancer patients were more likely to have a sexual partner (98.00% vs. 85.50%, *P* = 0.015), had lower BMI (22.51 ± 3.03 vs. 23.84 ± 3.60 kg/m², *P* = 0.017), and showed lower diabetes prevalence (14.00% vs. 45.50%, *P <*0.001). No significant differences were observed between groups in smoking history, hypertension status, or neutrophil counts in the validation population.

**Table 2:** Characteristics among female population ≥20 years of age from Shenzhen Second People’s Hospital (n = 250).

Characteristics	All participants (n =250)	Without breast cancer (n = 200)	With breast cancer (n = 50)	*P*-value
Age, years	52.14 ± 14.45	50.80 ± 14.77	57.54 ± 11.73	0.003
Marital status, %				0.015
Sexual partner	88.00	85.50	98.00	
Asexual	12.00	14.50	2.00	
Ever been pregnant, %				0.083
No	10.80	12.50	4.00	
Yes	89.20	87.50	96.00	
BMI, kg/m^2^	23.57 ± 3.53	23.84 ± 3.60	22.51 ± 3.03	0.017
Smoking history, %				0.616
Never	99.60	99.50	100.00	
Yes	0.40	0.50	0.00	
Hypertension, %				0.481
No	72.00	71.00	76.00	
Yes	28.00	29.00	24.00	
Diabetes, %				<0.001
No	60.80	54.50	86.00	
Yes	39.20	45.50	14.00	
Neutrophil counts,10^3^/µL	3.92 ± 1.25	3.87 ± 1.16	4.13 ± 1.54	0.199
Lymphocyte counts, 10^3^/µL	1.84 ± 0.64	2.00 ± 0.57	1.20 ± 0.50	<0.001
NLR	2.38 ± 1.30	2.01 ± 0.59	3.86 ± 2.10	<0.001

Mean ± SD for continuous variables: the *P*-value was calculated by the Student’s t-test; (%) for categorical variables: the *P*-value was calculated by the chi-square test. BMI, body mass index; NLR, neutrophil-to-lymphocyte ratio.

### 3.2 NLR and FBC: results from multivariate models

**Figure 2:**
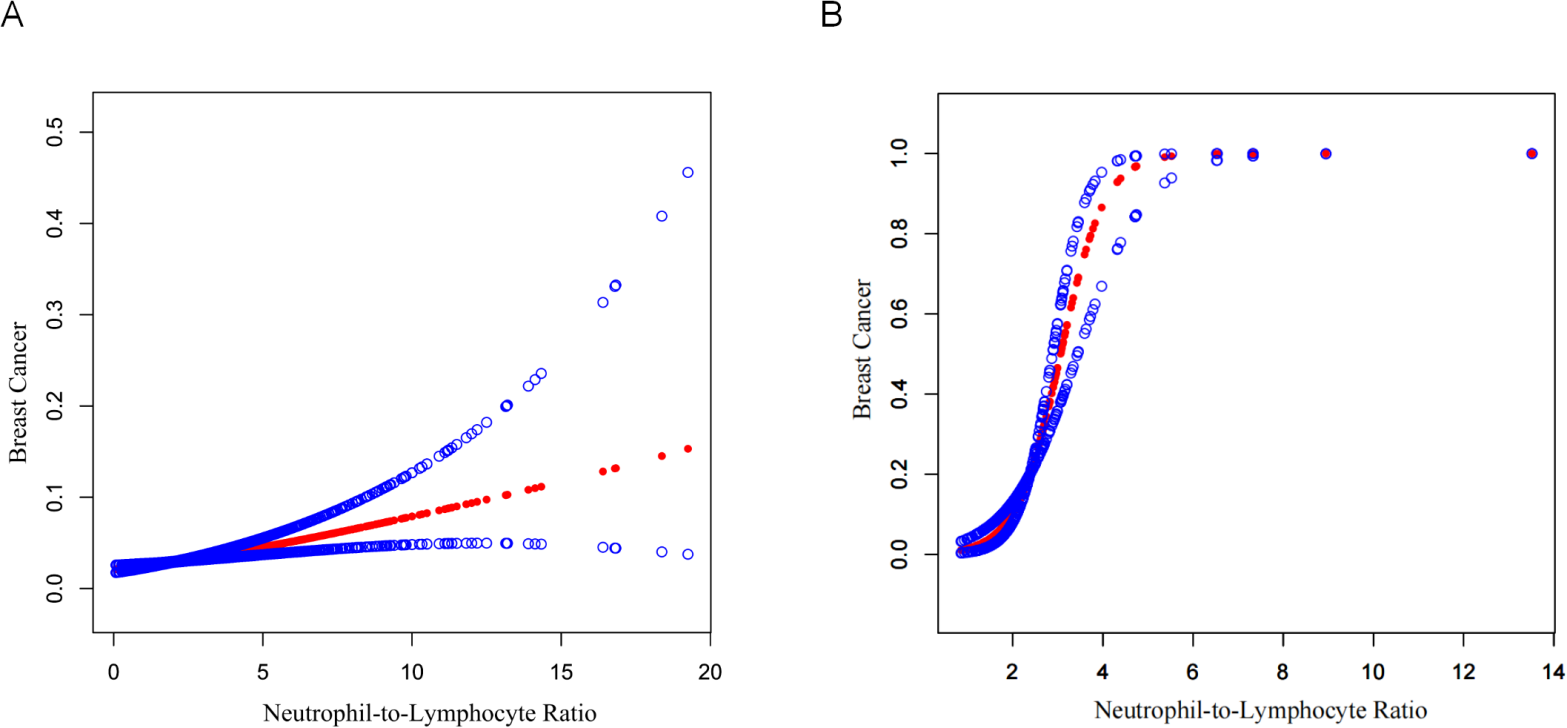
Curve fitting of NLR and FBC. **(A)** NHANES 2001–2018 cohort (N=15,313). A smooth curve fit between variables is shown by the solid red line, while the blue shaded regions indicate the 95% confidence interval around this fit. Age, race/ethnicity, education level, marital status, BMI, diabetes status, hypertension status, oral contraceptive use, age at menarche, ever been pregnant, and smoking history were adjusted. **(B)** External validation cohort from Shenzhen Second People’s Hospital (N=250). The curve demonstrates the validation of the NLR-breast cancer relationship in an independent Chinese population. The analysis was adjusted for age, marital status, BMI, diabetes status, hypertension status, pregnancy history, and smoking status.

**Table 3** is an illustration of NLR’s association with FBC. This study found a statistically substantial positive association between continuous NLR and FBC in the unadjusted model [OR = 1.22 (95% CI: 1.15, 1.29)] and partially adjusted model [OR = 1.14 (95% CI: 1.07, 1.21)]. After adjusting for covariates, for every unit elevation in NLR, the odds of FBC prevalence increased by 14% [OR = 1.14 (95% CI: 1.08, 1.22)]. When the NLR was divided into quartiles, the above association remained significant (all *P* for trend < 0.001). Participants in the highest quartile of NLR showed 67% increased odds of FBC prevalence [OR = 1.67 (95% CI: 1.24, 2.24)] compared to participants in the lowest quartile of NLR. Model fitness was assessed using AIC and BIC criteria for two NLR specifications and the multivariable-adjusted models demonstrated superior fit compared to unadjusted models. Specifically, for continuous NLR, Model I yielded AIC and BIC values of 4060.78 and 4076.05 respectively. These values decreased substantially to 3516.51 and 3562.33 after adjusting for demographic factors in Model II. Additional adjustment for clinical parameters in Model III led to a modest AIC reduction to 3492.83 but slightly increased BIC to 3622.65, suggesting a balance between model complexity and fit. Similar patterns were observed in categorical NLR analysis, supporting the consistency of our findings (**Table 4**). Additionally, the smoothed curve analysis further supported the positive association of NLR with FBC (**Figure 2A**).

**Table 3:** Multiple logistic regression analysis of NLR and breast cancer.

Outcome	Model I	Model II	Model III
	OR (95% CI, *P*)	OR (95% CI, *P*)	OR (95% CI, *P*)
Continuous NLR	1.22 (1.15, 1.29) *P* < 0.0001	1.14 (1.07, 1.21) *P* < 0.0001	1.14 (1.08, 1.22) *P* < 0.0001
Categories			
Q1 (0.97-1.31)	Reference	Reference	Reference
Q2 (1.56-1.79)	1.44 (1.07, 1.95), *P* = 0.0178	1.36 (1.00, 1.86), *P* = 0.0489	1.35 (0.99, 1.85), *P*= 0.0558
Q3 (2.05-2.36)	1.66 (1.24, 2.23), *P* = 0.0007	1.52 (1.12, 2.06), *P* = 0.0071	1.49 (1.10, 2.02), *P*= 0.0103
Q4 (2.82-3.87)	2.18 (1.64, 2.88), *P* < 0.0001	1.67 (1.24, 2.24), *P* = 0.0007	1.67 (1.24, 2.24), *P*= 0.0007
*P* for trend	<0.001	<0.001	<0.001

Model I: no covariates were adjusted. Model II: age and race/ethnicity were adjusted. Model III: age, race/ethnicity, education level, marital status, BMI, hypertension status, diabetes status, ever been pregnant, oral contraceptive use, age at menarche, and smoking history were adjusted. NLR, neutrophil-to-lymphocyte ratio; BMI, body mass index; Q means quartile; OR, odds ratio; 95% CI, 95% confidence interval.

**Table 4:** A model comparison using AIC and BIC criteria across different NLR specifications.

Specification	Model	AIC	BIC
Continuous NLR	Model I	4060.78	4076.05
	Model II	3516.51	3562.33
	Model III	3492.83	3622.65
Categories	Model I	4070.64	4101.18
	Model II	3523.62	3584.71
	Model III	3500.63	3645.72

Model I: no covariates were adjusted. Model II: age and race/ethnicity were adjusted. Model III: age, race/ethnicity, education level, marital status, BMI, hypertension status, diabetes status, ever been pregnant, oral contraceptive use, age at menarche, and smoking history were adjusted. AIC, Akaike Information Criterion; BIC, Bayesian Information Criterion; NLR, neutrophil-to-lymphocyte ratio.

External validation using the Shenzhen Second People’s Hospital dataset confirmed these findings (**Table 5**). The association between continuous NLR and breast cancer remained highly significant across all models, with ORs of 8.39 (95% CI: 4.40, 16.02, *P <*0.0001) in the unadjusted model, 8.08 (95% CI: 4.15, 15.73, *P <*0.0001) in the partially adjusted model, and 7.69 (95% CI: 3.72, 15.92, *P <*0.0001) in the fully adjusted model. Notably, the validation population demonstrated even stronger associations than the NHANES population, with substantially higher OR values. Categorical analysis showed participants in the highest NLR quartile (Q3) had significantly increased odds of breast cancer across all models [fully adjusted OR = 41.08 (95% CI: 8.09, 208.63), *P <*0.0001]. The model performance comparison (**Table 6**) indicated that continuous NLR specification provided better model fit than categorical specification in the validation dataset, with lower AIC values across all adjustment levels, further supporting the robustness of the NLR-breast cancer association. Furthermore, the smooth curve fitting analysis provided additional evidence for the positive relationship between NLR and FBC (**Figure 2B**).

**Table 5:** External validation of associations between NLR and breast cancer using the Shenzhen Second People’s Hospital data.

Outcome	Model I	Model II	Model III
	OR (95% CI, *P*)	OR (95% CI, *P*)	OR (95% CI, *P*)
Continuous NLR	8.39 (4.40, 16.02), *P <*0.0001	8.08 (4.15, 15.73), *P <*0.0001	7.69 (3.72, 15.92), *P <*0.0001
Categories			
Q1(0.85-1.80)	Reference	Reference	Reference
Q2(1.81-2.46)	4.93 (1.03, 23.54), *P*=0.0457	5.30 (1.09, 25.80), *P*=0.0388	6.30 (1.13, 35.05), *P*=0.0355
Q3(2.46-13.52)	35.10 (8.09, 152.20), *P*<0.0001	39.00 (8.73, 174.23), *P*<0.0001	41.08 (8.09, 208.63), P<0.0001
*P* for trend	<0.001	<0.001	<0.001

Model I: no covariates were adjusted. Model II: age and marital status were adjusted. Model III: age, marital status, BMI, hypertension status, diabetes status, ever been pregnant, and smoking history were adjusted. NLR, neutrophil-to-lymphocyte ratio; BMI, body mass index; Q means tertile; OR, odds ratio; 95% CI, 95% confidence interval.

**Table 6:** Comparison of AIC and BIC for continuous versus categorical NLR in the Shenzhen Second People’s Hospital validation dataset.

Specification	Model	AIC	BIC
Continuous NLR	Model I	156.47	163.52
	Model II	154.65	168.73
	Model III	139.81	171.51
Categories	Model I	197.85	208.41
	Model II	189.89	207.50
	Model III	167.13	202.34

Lower values of AIC and BIC indicate better model fit while accounting for model complexity.

Model I: no covariates were adjusted. Model II: age and marital status were adjusted. Model III: age, marital status, BMI, hypertension status, diabetes status, ever been pregnant, and smoking history were adjusted. AIC, Akaike Information Criterion; BIC, Bayesian Information Criterion; NLR, neutrophil-to-lymphocyte ratio.

### 3.3 Subgroup analysis of the correlation between NLR and FBC

To evaluate the consistency of the association of NLR with FBC, subgroup analyses and interaction tests were performed with stratification by age, race/ethnicity, education level, marital status, BMI, Hypertension, and diabetes. All tested variables showed no significant interaction effects, with all *P* for interaction values exceeding 0.05 (**Table 7**). The external validation analysis demonstrated remarkable stability of the association between NLR and breast cancer, with no significant interaction effects (all *P* for interaction > 0.05, **Table 8**).

**Table 7:** Subgroup analysis of the association between NLR and breast cancer.

Subgroup	Breast cancer OR (95% CI, *P*)	*P* for interaction
Age at interview, years		0.7716
< 60 years	1.15 (0.99, 1.32), *P* = 0.0651	
≥ 60 years	1.17 (1.10, 1.25), *P* < 0.0001	
Race/Ethnicity, %		0.3980
Non-Hispanic White	1.13 (1.05, 1.22), *P* = 0.0012	
Non-Hispanic Black	1.21 (1.04, 1.41), *P* = 0.0155	
Mexican American	1.24 (1.06, 1.45), *P* = 0.0073	
Other Race	1.34 (1.10, 1.63), *P* = 0.0031	
Education level, %		0.8856
Below high school	1.19 (1.05, 1.34), *P* = 0.0049	
High school	1.18 (1.07, 1.31), *P* = 0.0015	
College or above	1.15 (1.05, 1.26), *P* = 0.0024	
Marital status, %		0.3793
Sexual partner	1.13 (1.04, 1.24), *P* = 0.0069	
Asexual	1.20 (1.11, 1.29), *P* < 0.0001	
BMI, kg/m^2^		0.4114
< 25	1.19 (1.07, 1.32), *P* = 0.0014	
25-30	1.21 (1.108, 1.34), *P* < 0.0001	
≥ 30	1.10 (0.99, 1.23), *P* = 0.0809	
Hypertension, %		0.9341
No	1.17 (1.07, 1.27), *P* = 0.0005	
Yes	1.17 (1.08, 1.27), *P* = 0.0001	
Diabetes, %		0.8133
No	1.17 (1.10, 1.26), *P* < 0.0001	
Yes	1.15 (1.02, 1.30), *P* = 0.0190	

Age, race/ethnicity, education level, marital status, BMI, hypertension status, diabetes status, ever been pregnant, oral contraceptive use, age at menarche, and smoking history were adjusted. When conducting subgroup analyses, the subgroup factor itself was not adjusted for. BMI, body mass index; NLR, neutrophil-to-lymphocyte ratio; OR, odds ratio; 95% CI, 95% confidence interval.

**Table 8:** Subgroup analysis of the association between NLR and breast cancer from Shenzhen Second People’s Hospital.

Subgroup	Breast cancer OR (95% CI, *P*)	*P* for interaction
Age at interview, years		0.6560
< 60 years	6.91 (2.76, 17.28), *P* < 0.0001	
≥ 60 years	9.38 (2.97, 31.18), *P* = 0.0002	
BMI, kg/m^2^		0.7712
< 25	8.08 (3.33, 17.87), *P* < 0.0001	
≥ 25	9.75 (2.40, 39.58), *P* = 0.0014	
Hypertension, %		0.8747
No	8.21 (3.51, 19.21), *P* < 0.0001	
Yes	7.24 (1.98, 26.46), *P* = 0.0028	
Diabetes, %		0.3294
No	6.78 (3.17, 14.50), *P* < 0.0001	
Yes	17.52 (2.52, 121.95), *P* = 0.0038	

Age, marital status, BMI, hypertension status, diabetes status, ever been pregnant, and smoking history were adjusted. When conducting subgroup analyses, the subgroup factor itself was not adjusted for. BMI, body mass index; NLR, neutrophil-to-lymphocyte ratio; OR, odds ratio; 95% CI, 95% confidence interval.

## 4 Discussion

Our study represents the first investigation of the association between NLR and breast cancer in female participants in the U.S. female population aged ≥20 years using NHANES data. We identified a significant positive relationship between NLR and female breast cancer. This association remained consistent across different population characteristics, as evidenced by our subgroup analyses and interaction tests. To strengthen the validity of our conclusions, we conducted external validation using an independent clinical cohort, which successfully replicated our key findings and confirmed the positive association between elevated NLR levels and breast cancer prevalence. This validation across different study populations and methodological approaches provides compelling evidence for the reliability and clinical relevance of our results.

A landmark meta-analysis by Templeton including 100 studies with 40,559 patients demonstrated that elevated NLR was significantly associated with poor overall survival across various solid tumors [HR = 1.81, (95% CI: 1.67-1.97)]. The prognostic impact was most pronounced in mesothelioma [HR = 2.35, (95% CI: 1.89-2.92)], followed by pancreatic cancer [HR = 2.27, (95% CI: 1.01-5.14)], and renal cell carcinoma [HR = 2.22, (95% CI: 1.72-2.88)] (24). Furthermore, elevated NLR demonstrated significant associations with several clinicopathological features. Meta-analysis results revealed that high NLR was significantly correlated with distant metastasis [OR = 1.69, (95% CI: 1.10-2.59)], poor tumor differentiation [OR = 2.75, (95% CI: 1.19-6.36)] (25). These findings further validated the significant clinical utility of NLR as a tumor prognostic biomarker. Previous studies have examined the correlation between preoperative NLR and outcome in FBC patients, it has been widely studied as a prognostic marker in cancer patients on the grounds that it reflects the inflammatory response to tumor (26, 27). The epidemiological methods and target populations used in these studies varied. A recent meta‐analysis of 39 studies (including 17,079 FBC patients) suggested that an elevated NLR demonstrated a relationship with poorer OS [HR = 1.78, (95% CI: 1.49-2.13)] and DFS [HR = 1.60, (95% CI: 1.42-1.96)] among FBC patients (28). In a retrospective study, we revealed that among 266 triple-negative breast cancer (TNBC) patients who had radical surgical intervention after receiving sequential anthracycline and taxane-based neoadjuvant chemotherapy (NAC), high tumor-infiltrating lymphocyte (TIL) levels [OR = 4.28, (95% CI: 1.40-13.1)] and low NLR [OR = 5.51, (95% CI: 1.60-18.9)] and low platelet-to-lymphocyte ratio (PLR) demonstrated significant association with complete response (pCR) [OR = 3.29, (95% CI: 1.13-9.57)]. Notably, low NLR independently predicted pCR [OR = 6.59, (95% CI: 1.45-30.0)], which suggested it might be a useful surrogate indicator of TILs (26). In another survey of ER-negative breast cancer patients, an increased NLR was reported to be independently associated with late-stage recurrence [HR = 1.448, (95% CI: 1.168-1.795)] (27). In a tertiary care center, a retrospective cohort study suggested that the higher NLR was significantly correlated with poor clinical outcomes in advanced breast cancer [OR = 2.08, (95% CI: 1.032-4.193)]. This association might be attributed to increased inflammatory mediator production by advanced tumors. Alternatively, chronic inflammatory processes could elevate NLR and subsequently accelerate tumor progression and metastasis (29). These findings demonstrate that NLR serves as a cost-effective inflammatory marker for breast cancer management, predicting treatment response prediction, recurrence risk, and prognostic evaluation. Recent studies showed that systemic inflammatory markers were correlated with the prognosis in breast cancer patients treated with CDK4/6 inhibitors (30–33). In the CDK4/6 inhibitor treatment cohort, high baseline NLR (>2.98) demonstrated significant association with shorter progression-free survival (PFS) and OS, which was confirmed by multivariate cox regression analysis [HR = 2.38, (95% CI: 1.23-4.6)], suggesting NLR could functions as a predictive marker for breast cancer patients receiving CDK4/6 inhibitor therapy (30). Further studies revealed that NLR after CDK4/6 inhibitor treatment was an important prognostic indicator for advanced breast cancer, with patients having NLR≥1.58 or dNLR≥1.04 on day 1 of cycle 2 showing significantly worse OS and PFS. For patients with very high dNLR (≥2.00) after treatment initiation, the addition of CDK4/6 inhibitor to letrozole therapy showed no significant survival advantage (32). Therefore, NLR may serve as an auxiliary indicator to evaluate the efficacy of CDK4/6 inhibitor therapy in FBC patients.

In addition to its role as a prognostic indicator, the relationship between NLR and breast cancer also suggests potential therapeutic strategies targeting inflammatory pathways, such as employing IL-1β inhibition (such as canakinumab)has demonstrated promise in reducing cancer mortality, particularly in lung cancer (34). Recent findings indicate that IL-1β exacerbates myocardial injury in cancer patients receiving chemotherapy and immune checkpoint inhibitors (35). However, the IL-1β blocking agent canakinumab has been shown to effectively reduce major adverse cardiovascular events and cardiovascular mortality (36). Cancer patients face increased risk of cardiovascular complications associated with coagulopathy, myocarditis, and heart failure compared to the general population. The blockade of IL-1β not only mitigates cancer progression but also provides a protective barrier against cardiovascular complications, which is of vital importance in the current era of SARS-CoV-2 infection. Therefore, the pharmacological inhibition of IL-1β represents a promising therapeutic strategy worthy of further investigation in breast cancer management (34).

### 4.1 Potential mechanism

According to the literature, NLR within the grey zone of 2.3-3.0 may function as an early biomarker for pathological conditions such as malignance. Although the specific cut-off values vary among different cancer types, the general rule is that higher NLR is linked to shorter OS and DFS (37, 38). Bowen et al. analyzed 144 studies (45,905 gastrointestinal cancer patients) and found that the mean, median, and mode cutoffs for NLR were 3.4, 3.0, and 5.0, respectively, when patients’ NLR was higher than these cutoff values, it indicated poorer prognosis (39). Our research has confirmed that NLR is positively correlated with the prevalence of breast cancer, it is possible that the NLR represents two opposing inflammatory and immune pathways, but the exact pathological and physiological mechanisms are still unclear (40). Rudolf Virchow first discovered leucocytes within neoplastic tissues in 1863, establishing an association between inflammatory processes and cancer development, the systemic inflammatory process, mediated by chemokines, leads to changes in blood cell populations, including neutrophilia and lymphopenia (41). In the inflammatory microenvironment, chemokines released by cancer cells trigger neutrophil adhesion to blood vessel endothelium, followed by their migration through vessels into surrounding tissues (42). Chemokines entered the bloodstream, which interacted with CXCR-1 and CXCR-2 (G protein-coupled receptors) on neutrophils and facilitated their directed movement toward tumor sites (43, 44).

Neutrophils play a complex role in tumor development, both promoting tumor growth and inhibiting tumor progression (45). In 1999, Knaapen et al. demonstrated for the first time that neutrophils have a direct pro-carcinogenic effect in a study (46). Canli et al. found that neutrophil-generated reactive oxygen species (ROS) contributed to increased mutations during inflammatory intestinal carcinogenesis (47). In another study, neutrophil-derived ROS were found to amplify DNA damage during carcinogen exposure, thereby promoting tumorigenesis, in a model of chemical carcinogenesis in the lung (48). In anaplastic thyroid cancer, tumor-associated neutrophils maintained viability through altered oxidative mitochondrial metabolism while releasing neutrophil extracellular traps (NETs) to promote cancer cell proliferation (49). Neutrophils promoted tumor metastasis and growth through multiple mechanisms including forming cell clusters (50), establishing pre-metastatic niches (51), immune suppression (52), releasing NETs (53), metabolic reprogramming (54), and tissue-specific modifications during the metastatic process (55). Recent years have witnessed growing interest in the anti-tumor effects of lymphocytes in the tumor microenvironment. Increased TILs indicated a better prognosis for the cancer patient (56). Firstly, TILs containing cytotoxic T cells and NK cells could directly recognize and kill tumor cells. They released cytotoxic molecules such as perforin and granzymes, which induce apoptosis of tumor cells (52). Secondly, TILs secreted various cytokines, such as Interferon-γ (IFN-γ) and TNF-α, which could suppress tumor angiogenesis and compromise tumor metabolic supply, and inhibited tumor-associated immunosuppressive cells, such as myeloid-derived suppressor cells (MDSC) and Treg, to restore anti-tumor immune function (57).

### 4.2 Strengths and limitations of this study

Our investigation presents several key strengths. Firstly, the investigation was based on a large, representative cohort. Secondly, it incorporated multiple potential confounding variables, including socio-demographic characteristics, reproductive health conditions, diabetes, and blood pressure, to strengthen result validity. Thirdly, we conducted external validation using an independent clinical dataset, which enhanced the robustness and generalizability of our findings across different populations and healthcare settings. Finally, this research was the first to exploring the association between NLR and breast cancer using the NHANES database. Nevertheless, several limitations warrant consideration. First, the self-reported nature of the NHANES questionnaire restricted our access to detailed pathological and molecular subtype data. Second, the cross-sectional study design precluded causal inference, suggesting the need for future longitudinal studies with larger cohorts. In addition, breast cancer data in our study were obtained through self-reporting rather than clinical assessments, potentially introducing recall bias. Furthermore, while our external validation study strengthened the evidence, it was limited by a relatively small sample size and single-center design, which may restrict the generalizability to broader clinical populations. The validation dataset also had limited covariate information compared to the NHANES data, potentially affecting the comprehensiveness of adjusted analyses. Finally, despite we adjusted for several potential covariates, the potential influence of additional unmeasured confounding factors could not be completely eliminated.

## 5 Conclusion

Our study found a positive association between elevated NLR and FBC, which was validated in an independent clinical sample, confirming the robustness of our findings. To strengthen the clinical utility of NLR as a prognostic biomarker in breast cancer, future large-scale prospective studies should focus on exploring the associations between NLR and breast cancer molecular subtypes and determining whether a causal relationship exists between these factors.

## Data Availability

The raw data supporting the conclusions of this article will be made available by the authors, without undue reservation.
